# Valuing infertility treatment: Why QALYs are inadequate, and an alternative approach to cost-effectiveness thresholds

**DOI:** 10.3389/fmedt.2022.1053719

**Published:** 2022-12-23

**Authors:** Elena Keller, Georgina M. Chambers

**Affiliations:** National Perinatal Epidemiology and Statistics Unit, Centre for Big Data Research in Health, UNSW Sydney, Sydney, NSW, Australia

**Keywords:** infertility treatment, quality-adjusted life-year (QALY), cost-benefit analysis (CBA), discrete choice experiment (DCE), willingness-to-pay (WTP), health technology assessment (HTA), value of a statistical baby (VSB)

## Introduction

The ability to reproduce is the most fundamental of all human activities, and thus the inability to do so (infertility) is acknowledged as a disease by key international bodies such as the World Health Organization and the United Nations (1). Infertility is an increasingly prevalent global health issue with >180 million people affected (2), with a similar prevalence among high- and low-income countries (3). The trend to later childbearing, increasing rates of obesity and some sexually transmitted infections, and the well-documented decline in sperm counts worldwide are largely responsible (4–6). While infertility is not a life-threatening disease, it can cause significant long-term psychological suffering, stigmatization, and violence towards women (2, 7).

Medically assisted infertility treatments allow many affected individuals to have children. More traditional forms of infertility treatment, such as ovulation induction and artificial insemination, are still widely used. However, the treatment of infertility has been revolutionized over the last forty years with the advent of assisted reproductive technologies (ART), such as *in vitro* fertilization (IVF) where fertilization of a woman's eggs occurs in a laboratory and the resulting embryo(s) is transferred into the uterus. Up to 9% of children in some countries are now conceived using ARTs (8), with >2 million ART treatment cycles performed each year (9).

Infertility treatment is expensive, particularly ARTs (∼US$12,000/cycle), with women often needing many cycles to achieve pregnancy, if at all. The value of the global infertility treatment market is estimated to reach US$27 billion by 2026 (10), representing a significant economic burden to healthcare systems, governments, third-party insurers, and patients.

There is arguably no other medical treatment that exhibits such varying arrangements for funding by governments and third parties as ART. In the latest global survey of ART practices and policies undertaken by the International Federation of Fertility Societies, fewer than half of the 85 countries surveyed reported any type of financial support for ART treatment, and only 17 offered full reimbursement (11). If funded, most programs use eligibility criteria (e.g., age, marital status) and apply limits on the number of treatment cycles financed (12–20). The consequence of this is unequal and inequitable access to ARTs, with a significant reliance on out-of-pocket funding (21).

Arguably, one of the key reasons for such variation in funding arrangements is the inadequacy of traditional health technology assessment (HTA) methods to capture the “value” of infertility treatments. Indeed, the most appropriate method for capturing the value of future parenthood has long been a challenge for economists (22). Typically, a cost-utility analysis (CUA) - which is the mainstay of HTA methods - reports outcomes in terms of additional cost per quality-adjusted life-year (QALY). The QALY is a composite measure that captures health-related quality of life (HRQoL) and life expectancy, the aim of which is to generate a single metric that allows direct comparisons of benefit across dissimilar health interventions.

However, as we will argue here, QALYs are not well suited to reflect the value associated with a new life generated using infertility treatment, and thus do not adequately inform decision-makers regarding the opportunity cost of allocating healthcare funds to ARTs compared to alternate health services. Instead, we argue that, in the absence of accepted preference-based utility instruments that go beyond HRQoL, the use of cost-effectiveness/willingness-to-pay (WTP) values for the birth of a baby within a cost-benefit analysis (CBA) framework is more appropriate for assessing value for money of infertility treatment.

## How are infertility treatments evaluated economically

Because of the challenges associated with QALYs in the context of infertility treatment, the majority of cost-effectiveness analyses use clinical outcome metrics such as live births or pregnancies and report incremental cost-effectiveness ratios based on the additional cost incurred to achieve an additional live birth or pregnancy (23–62). While such studies allow conclusions about the comparative cost-effectiveness within the narrow scope of infertility treatment, the WTP threshold for a child created through infertility treatments is unknown and thus it is unclear whether a treatment represents good value for money.

Using QALYs to measure the utility of infertility treatment outcomes within a CUA framework is uncommon and inconsistently applied (63). Some studies have considered QALYs of the mother (64), some of both parents (65, 66), and some of the children born through infertility treatment (67). While the use of QALYs to measure outcomes is conceptually appealing there are several issues in the context of infertility treatment that limit their applicability.

## QALYs: Creation of new life vs. changes in HRQoL of existing lives

Infertility treatment is a medical intervention to treat a disease. However, it is unique because unlike most medical interventions which are assessed based on their ability to improve, extend, or save an existing life, infertility treatment is judged based on its ability to create a new life. As argued by Devlin (68), QALYs were designed to capture improvements in patients, not to value new life. Utility weights, which reflect individuals’ preferences for specific health states, only exist for health states of an existing life (69–71). Simply applying lifetime QALYs to a new life for use in a CUA is not appropriate because additional lives are not improvements in health and preventing someone's death is not the same as creating their life. It is not possible to improve the quality of life of someone who has not been conceived by conceiving them (68).

In line with this argument, Luyten (72) recently proposed guidelines for inclusion of QALYs for health interventions that affect future lives, including contraception, abortion, disease screening and infertility treatment. According to these guidelines, QALYs of future lives should only be included if these lives would exist independent of the treatment choice. Since the creation of life in the context of infertility treatment is only possible for women who choose to undergo infertility treatment, QALYs of these future lives should not be considered in HTA of infertility treatment. While it can be perceived as inconsistent that QALYs are a valid metric for assessing perinatal interventions (e.g., perinatal screening to avoid disease) (73–75) but are not adequate for assessing infertility treatment, it is important to differentiate the timing and objective of the decision/choice to undergo the intervention when evaluating it. In the case of perinatal interventions and screening, the child is “necessary/assumed” for the healthcare service, and thus health impacts for that child should be counted. In contrast, infertility treatment and other reproductive interventions affect the “potential” to have a child and thus health impacts should not be counted.

Furthermore, to be consistent, the application of QALYs would need to be equally applied to interventions that increase fertility (e.g., infertility treatment) and those that reduce fertility (e.g., contraception). If arguing that the number of QALYs a child born through infertility treatment would experience should be included in economic evaluations, then the number of QALYs lost due to preventing the birth of a child should also be considered (72). Such an approach to valuing potential life is conceptually flawed, ethically problematic, and has not been adopted within a HTA framework (63).

## Parenthood impacts quality of life domains beyond health

Certainly, there is scope to use QALYs to value the reduction in psychological distress in would-be parents by creating a new life using infertility treatment. However, infertile couples who resolve their childlessness report high levels of life satisfaction and self-esteem, but not depressive symptoms, the latter of which are more likely to be captured by the domains included in common QALY instruments such as the EQ-5D (76–78).

While QALYs capture changes in HRQoL, arguably most of the benefits (and suffering) derived from having a child are not health-related, but has more to do with wellbeing, living a meaningful life and achieving life goals (68–70). However, infertility treatment is a medical intervention that competes for healthcare funding within a HTA framework and is thus further disadvantaged in terms of the utility/disutility captured by QALYs. The need to capture broader benefits of healthcare intervention, such as wellbeing, is increasingly being recognized (79) and has led to initiatives such as the *Extending the QALY* project which aims to develop a broader QALY measure (EQ-Health and Wellbeing, EQ-HWB) (80).

## Cost-benefit framework as alternative to assess value for money

In the absence of broader validated preference-based measures (e.g., EQ-HWB) that are applicable to unborn life, CBA is a more appropriate method for assessing the value for money of infertility treatments (68–70). In a CBA both costs and benefits of an intervention are monetized and, therefore, can be compared directly and the net monetary benefit (NMB) derived (NMB = benefits–costs). Interventions for which the NMB is positive represent good value for money as benefits outweigh costs. To monetize the benefits of an intervention, WTP values for a child created through infertility treatment are elicited (based on stated preferences in hypothetical questions) or observed (based on market behavior). However, while the latter is often preferred, the derivation of WTP values for infertility treatment based on observed market behavior is limited because price signals in healthcare markets are often highly distorted by insurance and confounded by the ability to pay (81). Alternatively, WTP values can be derived using hypothetical stated-preference methods, the most common being discrete choice experiments (DCEs). In these survey-based experiments, respondents choose their preferred treatment scenario in a sequence of choice tasks, where each treatment is described in terms of its key characteristics. In the case of infertility treatment, these characteristics would include success rates (i.e., chance of a live birth) and cost (i.e., cost of one ART cycle). Thereby respondents implicitly reveal their trade-off between the success rate and costs. Respondent choices capture a much wider utility function than HRQoL, including the intrinsic value of a newly created life and, if required equity and ethical preferences, thus overcoming a number of limitations of QALYs. The marginal rate of substitution then allows the value for a statistical baby (VSB) to be derived (82), much in the same way that a value of a statistical life (VSL) is derived. The VSL is an established economic concept used to inform policy in a variety of areas including public resource allocation to transport and environmental investment (83).

A DCE conducted among taxpayers in Australia illustrates this approach: Botha (84) reported that an average taxpayer was willing to pay $2.23 per year for a 1% improvement in the chance of having a baby per infertility treatment cycle. Applying a similar concept as the VSL, the VSB can be derived as the WTP value divided by the change in the treatment success rate (i.e., $2.23/1%) resulting in a WTP per statistical baby of $223 per taxpayer. This VSB could then be used in a CBA to monetize infertility treatment success (i.e., the birth of a baby). Comparing this value to the cost of infertility treatment for the creation of one baby establishes whether treatment represents value for money. By extension, the value for money of different eligibility criteria for public funding (e.g., age and number of ART treatment cycles) can be evaluated to inform resource allocation. That is, the number of ART cycles funded would be limited to those where the cost of treatment does not exceed the taxpayers’ WTP for a statistical baby.

While introducing a CBA framework in HTA assessments moves closer to capturing the true value of reproductive interventions, such as infertility treatment, and explicitly allows the NMB to be derived, decision rules would still be needed to decide how many QALYs from other health interventions should be forgone to fund such interventions. However, given the increasing recognition that many healthcare interventions have significant non-health benefits, this is perhaps inevitable (85).

Indeed, the increasing reliance on HTA for decision-making in health systems globally and the increasing recognition of the limitations of QALYs and current WTP thresholds requires alternative approaches to assess value-for-money by HTA organizations. There are currently no specific best-practice guidelines for cases where QALYs or current tools such as the EQ-5D are not fit-for-purpose. For instance, a recent NICE methods review found that there “is currently no guidance on what to do if EQ-5D is not available in the clinical trials or the literature, and it is not possible to map from another measure to EQ-5D” (86). In general, the NICE guidelines manual advises the use of a CUA framework where QALYs are applicable or, otherwise, a cost-effectiveness framework using cases averted or a disease-specific outcome as effectiveness measure (87). A criticism of CBA is that they do not consider equity and distributional concerns (88, 89), however this is also true for CUA in its standard form, and, if DCEs are used to derive the WTP thresholds, equity considerations can be implicitly incorporated. Rather, a key reason why HTA agencies reject CBAs seems to be the lack of guidelines on how results of a CEA, CUA and CBA can be compared to make resource allocation decisions (90).

There are a growing number of exceptions and exclusions to using the standard HTA framework. A case in point is the emergence of high-cost disease modifying therapies for rare diseases where WTP thresholds of up to $500,000 are considered acceptable (73, 88). Other concerns about the standardized use of QALYs include that they disadvantage those who are severely ill, disabled and are at the end-of-life, as well as not incorporating equity and distributional concerns in their standard form. A number of authors have proposed modifications to the current QALY-based HTA framework or proposed a wider use of cost-consequence studies (89–92). Such limitations have also prompted reviews of current HTA frameworks including in Australia (93) and the UK (94, 95).

## Conclusion

Infertility treatment competes with other medical interventions for healthcare funds within a HTA framework but is uniquely assessed based on its ability to create life, rather than improve, extend, or save existing life. CUAs measuring treatment outcomes in QALYs are not fit-for-purpose to evaluate infertility treatments. Therefore, appropriate methods that holistically capture health *and* non-health benefits to assess the cost-effectiveness of infertility treatments are required. McIntosh (96) described how WTP values for treatment outcomes derived from a DCE can be used in a CBA over 15 years ago. However, even though the number of DCEs have increased significantly over this time, their results are rarely applied in a policy-relevant context. It is increasingly being recognized that broader benefits beyond health should be considered when assessing value for money in healthcare. Therefore, it is important that the health economic research agenda develops standards and best-practice guidelines for the incorporation of DCEs and CBA into HTA (97), including how CBA results should be compared to those obtained using CUAs to allocate healthcare funding.

## References

[1] World Health Organization. Infertility Geneva: World Health Organization (2020). Available at: https://www.who.int/news-room/fact-sheets/detail/infertility (Accessed 14 September, 2020–12 November 2021).

[2] Inhorn MC Patrizio P. Infertility around the globe: new thinking on gender, reproductive technologies and global movements in the 21st century. Hum Reprod Update. (2015) 21(4):411–26. 10.1093/humupd/dmv016 25801630

[3] Mascarenhas MN Flaxman SR Boerma T Vanderpoel S Stevens GA. National, regional, and global trends in infertility prevalence since 1990: a systematic analysis of 277 health surveys. PLoS Med. (2012) 9(12):e1001356. 10.1371/journal.pmed.1001356 23271957 PMC3525527

[4] Homan GF Davies M Norman R. The impact of lifestyle factors on reproductive performance in the general population and those undergoing infertility treatment: a review. Hum Reprod Update. (2007) 13(3):209–23. 10.1093/humupd/dml056 17208948

[5] Tough S Tofflemire K Benzies K Fraser-Lee N Newburn-Cook C. Factors influencing childbearing decisions and knowledge of perinatal risks among Canadian men and women. Matern Child Health J. (2007) 11(2):189–98. 10.1007/s10995-006-0156-1 17237994

[6] Levine H Jørgensen N Martino-Andrade A Mendiola J Weksler-Derri D Mindlis I Temporal trends in sperm count: a systematic review and meta-regression analysis. Hum Reprod Update. (2017) 23(6):646–59. 10.1093/humupd/dmx022 28981654 PMC6455044

[7] Luk BH-K Loke AY. The impact of infertility on the psychological well-being, marital relationships, sexual relationships, and quality of life of couples: a systematic review. J Sex Marital Ther. (2015) 41(6):610–25. 10.1080/0092623X.2014.958789 25211377

[8] European IVF Monitoring Consortium ftESoHR, Embryology, Wyns C De Geyter C Calhaz-Jorge C Kupka MS ART in Europe, 2018: results generated from European registries by eshre†. Hum Reprod Open. (2022) 2022(3):1–20. 10.1093/hropen/hoac022 PMC9252765 35795850

[9] Chambers GM Dyer S Zegers-Hochschild F de Mouzon J Ishihara O Banker M International committee for monitoring assisted reproductive technologies world report: assisted reproductive technology, 2014†. Hum Reprod. (2021) 36(11):2921–34. 10.1093/humrep/deab198 34601605

[10] Sumant O Joshi K. Fertility services market by procedure (IVF with ICSI, IUI, IVF without ICSI, surrogacy, and others) and service (Fresh non-donor, frozen non-donor, egg and embryo banking, fresh donor, and frozen donor): global opportunity analysis and industry forecast, 2019–2026. Portland, Oregon (2019) LI_184557.

[11] International Federation of Fertility Societies. International federation of fertility societies’ surveillance (IFFS) 2019: global trends in reproductive policy and practice, 8th edition. Glob Reprod Health. (2019) 4(1):e29. 10.1097/grh.0000000000000029

[12] Chambers GM Keller E Choi S Khalaf Y Crawford S Botha W Funding and public reporting strategies for reducing multiple pregnancy from fertility treatments. Fertil Steril. (2020) 114(4):715–21. 10.1016/j.fertnstert.2020.08.1405 33040980

[13] Berg Brigham K Cadier B Chevreul K. The diversity of regulation and public financing of IVF in Europe and its impact on utilization. Hum Reprod. (2013) 28(3):666–75. 10.1093/humrep/des418 23223400

[14] Fertility Europe, European Society of Human Reproduction and Embryology. A policy audit on fertility: analysis of 9 Eu countries: March 2017. Brussels (2017).

[15] Bundesministerium für Gesundheit und Frauen. Wir Möchten Ein Baby: Information Über Kostenübernahme Für Medizinisch Unterstützte Fortpflanzung Durch Den IVF-Fonds. Vienna (2017).

[16] International Federation of Fertility Societies. IFFS surveillance 2016. Glob Reprod Health. (2016) 1:e1. 10.1097/GRH.0000000000000001

[17] Australian Government Department of Health. Medicare benefits schedule book: Operating from 1 August 2019. Canberra: Commonwealth of Australia (2019).

[18] Northern Region Fertility Service (NRFS). Detail on eligibility for publicly funded fertility services. Auckland (2018).

[19] Auckland District Health Board National Women's Health. Our services: fertility: public funding Auckland: National Women's Health (2019). Available at: https://nationalwomenshealth.adhb.govt.nz/our-services/fertility/public-funding/ (Accessed 6 December, 2019).

[20] International Federation of Fertility Societies. IFFS surveillance 2019: global trends in reproductive policy and practice, 8th edition. Available at: https://www.iffsreproduction.org/page/surveillance (Accessed June, 2020).

[21] Chambers GM Hoang VP Sullivan EA Chapman MG Ishihara O Zegers-Hochschild F The impact of consumer affordability on access to assisted reproductive technologies and embryo transfer practices: an international analysis. Fertil Steril. (2014) 101(1):191–8. 10.1016/j.fertnstert.2013.09.005 24156958

[22] Myers ER. How should we estimate the cost-effectiveness of interventions that affect reproduction? Med Decis Making. (2015) 35(7):812–4. 10.1177/0272989/15602227 26296618

[23] Pashayan N Lyratzopoulos G Mathur R. Cost-effectiveness of primary offer of IVF vs. primary offer of IUI followed by IVF (for IUI failures) in couples with unexplained or mild male factor subfertility. BMC Health Serv Res. (2006) 6(1):80. 10.1186/1472-6963-6-80 16796733 PMC1543624

[24] Goverde AJ McDonnell J Vermeiden JPW Schats R Rutten FFH Schoemaker J. Intrauterine insemination or in-vitro fertilisation in idiopathic subfertility and male subfertility: a randomised trial and cost-effectiveness analysis. Lancet. (2000) 355(9197):13–8. 10.1016/S0140-6736(99)04002-7 10615885

[25] Braam SC Ho VNA Pham TD Mol BW van Wely M Vuong LN. In-vitro maturation versus IVF: a cost-effectiveness analysis. Reprod Biomed Online. (2021) 42(1):143–9. 10.1016/j.rbmo.2020.09.022 33132059

[26] Moolenaar LM Cissen M de Bruin JP Hompes PGA Repping S van der Veen F Cost-effectiveness of assisted conception for male subfertility. Reprod Biomed Online. (2015) 30(6):659–66. 10.1016/j.rbmo.2015.02.006 25900905

[27] van Loendersloot LL Moolenaar LM Mol BWJ Repping S van der Veen F Goddijn M. Expanding reproductive lifespan: a cost-effectiveness study on oocyte freezing. Hum Reprod. (2011) 26(11):3054–60. 10.1093/humrep/der284 21896545

[28] Mersereau JE Plunkett BA Cedars MI. Preimplantation genetic screening in older women: a cost-effectiveness analysis. Fertil Steril. (2008) 90(3):592–8. 10.1016/j.fertnstert.2007.07.1307 18001724

[29] van Eekelen R Eijkemans MJ Mochtar M Mol F Mol BW Groen H Cost-effectiveness of medically assisted reproduction or expectant management for unexplained subfertility: when to start treatment? Hum Reprod. (2020) 35(9):2037–46. 10.1093/humrep/deaa158 32876323 PMC7550266

[30] Murugappan G Ohno MS Lathi RB. Cost-Effectiveness analysis of preimplantation genetic screening and in vitro fertilization versus expectant management in patients with unexplained recurrent pregnancy loss. Fertil Steril. (2015) 103(5):1215–20. 10.1016/j.fertnstert.2015.02.012 25772770

[31] Bhatti T Baibergenova A. A comparison of the cost-effectiveness of in vitro fertilization strategies and stimulated intrauterine insemination in a Canadian health economic model. J Obstet Gynaecol Can. (2008) 30(5):411–20. 10.1016/S1701-2163(16)32826-2 18505665

[32] Collins SC Xu X Mak W. Cost-effectiveness of preimplantation genetic screening for women older than 37 undergoing in vitro fertilization. J Assist Reprod Genet. (2017) 34(11):1515–22. 10.1007/s10815-017-1001-8 28748493 PMC5699982

[33] Facadio Antero M Singh B Pradhan A Gornet M Kearns WG Baker V Cost-effectiveness of preimplantation genetic testing for aneuploidy for fresh donor oocyte cycles. F S Reports. (2021) 2(1):36–42. 10.1016/j.xfre.2020.11.005 34223271 PMC8244284

[34] Tjon-Kon-Fat RI Bensdorp AJ Bossuyt PMM Koks C Oosterhuis GJE Hoek A Is IVF - served two different ways - more cost-effective than IUI with controlled ovarian hyperstimulation? Hum Reprod. (2015) 30(10):2331–9. 10.1093/humrep/dev193 26269539

[35] Moolenaar LM Nahuis MJ Hompes PG van der Veen F Mol BWJ. Cost-effectiveness of treatment strategies in women with PCOS who do not conceive after six cycles of clomiphene citrate. Reprod Biomed Online. (2014) 28(5):606–13. 10.1016/j.rbmo.2014.01.014 24656560

[36] Philips Z Barraza-Llorens M Posnett J. Evaluation of the relative cost-effectiveness of treatments for infertility in the UK. Hum Reprod. (2000) 15(1):95–106. 10.1093/humrep/15.1.95 10611196

[37] Hollingsworth B Harris A Mortimer D. The cost effectiveness of intracyctoplasmic sperm injection (ICSI). J Assist Reprod Genet. (2007) 24(12):571. 10.1007/s10815-007-9175-0 18008157 PMC3454996

[38] Lee M Lofgren KT Thomas A Lanes A Goldman R Ginsburg ES The cost-effectiveness of preimplantation genetic testing for aneuploidy in the United States: an analysis of cost and birth outcomes from 158,665 in vitro fertilization cycles. Am J Obstet Gynecol. (2021) 225(1):55.e1–55.e17. 10.1016/j.ajog.2021.01.021 33539823

[39] Lee E Costello MF Botha WC Illingworth P Chambers GM. A cost-effectiveness analysis of preimplantation genetic testing for aneuploidy (PGT-A) for up to three complete assisted reproductive technology cycles in women of advanced maternal age. Aust N Z J Obstet Gynaecol. (2019) 59(4):573–9. 10.1111/ajo.12988 31106861

[40] Bordewijk EM Weiss NS Nahuis MJ Bayram N van Hooff MHA Boks DES Gonadotrophins versus clomiphene citrate with or without IUI in women with normogonadotropic anovulation and clomiphene failure: a cost-effectiveness analysis. Hum Reprod. (2019) 34(2):276–84. 10.1093/humrep/dey359 30576539

[41] Sykes D Out HJ Palmer SJ van Loon J. The cost-effectiveness of IVF in the UK: a comparison of three gonadotrophin treatments. Hum Reprod. (2001) 16(12):2557–62. 10.1093/humrep/16.12.2557 11726574

[42] van Loendersloot LL Moolenaar LM van Wely M Repping S Bossuyt PM Hompes PGA Cost-effectiveness of single versus double embryo transfer in IVF in relation to female age. Eur J Obstet Gynecol Reprod Biol. (2017) 214:25–30. 10.1016/j.ejogrb.2017.04.031 28460276

[43] Le KD Vuong LN Ho TM Dang VQ Pham TD Pham CT A cost-effectiveness analysis of freeze-only or fresh embryo transfer in IVF of non-pcos women. Hum Reprod. (2018) 33(10):1907–14. 10.1093/humrep/dey253 30239784

[44] Polinder S Heijnen EMEW Macklon NS Habbema JDF Fauser BJCM Eijkemans MJC. Cost-effectiveness of a mild compared with a standard strategy for IVF: a randomized comparison using cumulative term live birth as the primary endpoint. Hum Reprod. (2007) 23(2):316–23. 10.1093/humrep/dem372 18033807

[45] Fiddelers AAA Dirksen CD Dumoulin JCM van Montfoort APA Land JA Janssen JM Cost-effectiveness of seven IVF strategies: results of a markov decision-analytic model. Hum Reprod. (2009) 24(7):1648–55. 10.1093/humrep/dep041 19318703

[46] van Tilborg TC Oudshoorn SC Eijkemans MJC Mochtar MH van Golde RJT Hoek A Individualized fsh dosing based on ovarian reserve testing in women starting IVF/ICSI: a multicentre trial and cost-effectiveness analysis. Hum Reprod. (2017) 32(12):2485–95. 10.1093/humrep/dex321 29121350

[47] Fiddelers AAA van Montfoort APA Dirksen CD Dumoulin JCM Land JA Dunselman GAJ Single versus double embryo transfer: cost-effectiveness analysis alongside a randomized clinical trial. Hum Reprod. (2006) 21(8):2090–7. 10.1093/humrep/del112 16613886

[48] Dixon S Faghih Nasiri F Ledger W Lenton E Duenas A Sutcliffe P Cost-effectiveness analysis of different embryo transfer strategies in England. BJOG: Int J Obstet Gynaecol. (2008) 115(6):758–66. 10.1111/j.1471-0528.2008.01667.x 18355368

[49] Groen H Tonch N Simons AHM van der Veen F Hoek A Land JA. Modified natural cycle versus controlled ovarian hyperstimulation IVF: a cost-effectiveness evaluation of three simulated treatment scenarios. Hum Reprod. (2013) 28(12):3236–46. 10.1093/humrep/det386 24166594

[50] Vitek WS Galárraga O Klatsky PC Robins JC Carson SA Blazar AS. Management of the first in vitro fertilization cycle for unexplained infertility: a cost-effectiveness analysis of split in vitro fertilization-intracytoplasmic sperm injection. Fertil Steril. (2013) 100(5):1381–8.e1. 10.1016/j.fertnstert.2013.06.035 23876534 PMC4503359

[51] van Hoogenhuijze NE van Eekelen R Mol F Schipper I Groenewoud ER Traas MAF Economic evaluation of endometrial scratching before the second IVF/ICSI treatment: a cost-effectiveness analysis of a randomized controlled trial (scratch trial). Hum Reprod. (2022) 37(2):254–63. 10.1093/humrep/deab261 34864993 PMC8804332

[52] Luo L Chen M Wen Y Zhang L Zhou C Wang Q. Pregnancy outcome and cost-effectiveness comparisons of artificial cycle-prepared frozen embryo transfer with or without gnrh agonist pretreatment for polycystic ovary syndrome: a randomised controlled trial. BJOG: Int J Obstet Gynaecol. (2021) 128(4):667–74. 10.1111/1471-0528.16461 32783367

[53] Crawford NM Sahay KM Mersereau JE. Mild stimulation versus conventional IVF: a cost-effectiveness evaluation. Open J Obstet Gynecol. (2016) 6(3):9. 10.4236/ojog.2016.63023

[54] Fiddelers AAA Severens JL Dirksen CD Dumoulin JCM Land JA Evers JLH. Economic evaluations of single- versus double-embryo transfer in IVF. Hum Reprod Update. (2006) 13(1):5–13. 10.1093/humupd/dml053 17099208

[55] Daya S Ledger W Auray JP Duru G Silverberg K Wikland M Cost-effectiveness modelling of recombinant FSH versus urinary FSH in assisted reproduction techniques in the UK. Hum Reprod. (2001) 16(12):2563–9. 10.1093/humrep/16.12.2563 11726575

[56] Lukassen HGM Braat DD Wetzels AMM Zielhuis GA Adang EMM Scheenjes E Two cycles with single embryo transfer versus one cycle with double embryo transfer: a randomized controlled trial. Hum Reprod. (2005) 20(3):702–8. 10.1093/humrep/deh672 15618254

[57] Bühler K Roeder C Schwarze J-E Lispi M Allignol A Falla E Cost-effectiveness analysis of recombinant human follicle-stimulating hormone alfa (R-HFSH) and urinary highly purified menopausal gonadotropin (HMG) based on data from a large German registry. Best Pract Res Clin Obstet Gynaecol. (2022). 10.1016/j.bpobgyn.2022.02.002 35304097

[58] Bordewijk EM Weiss NS Nahuis MJ Kwee J Lambeek AF van Unnik GA Gonadotrophins or clomiphene citrate in women with normogonadotropic anovulation and cc failure: does the endometrium matter? Hum Reprod. (2020) 35(6):1319–24. 10.1093/humrep/deaa052 32585686 PMC7316496

[59] Little SE Ratcliffe J Caughey AB. Cost of transferring one through five embryos per in vitro fertilization cycle from various payor perspectives. Obstet Gynecol. (2006) 108(3):593–601. 10.1097/01.aog.0000230534.54078.b3 16946220

[60] Eijkemans MJC Kersten FAM Lintsen AME Hunault CC Bouwmans CAM Roijen LH Cost-effectiveness of ‘immediate IVF’ versus ‘delayed IVF’: a prospective study. Hum Reprod. (2017) 32(5):999–1008. 10.1093/humrep/dex018 28204519

[61] Suchartwatnachai C Wongkularb A Srisombut C Choktanasiri W Chinsomboon S Rojanasakul A. Cost-effectiveness of IVF in women 38 years and older. Int J Gynaecol Obstet. (2000) 69(2):143–8. 10.1016/S0020-7292(99)00215-5 10802082

[62] Griffiths A Dyer SM Lord SJ Pardy C Fraser IS Eckermann S. A cost-effectiveness analysis of in-vitro fertilization by maternal age and number of treatment attempts. Hum Reprod. (2010) 25(4):924–31. 10.1093/humrep/dep418 20106837

[63] Goldhaber-Fiebert JD Brandeau ML. Evaluating cost-effectiveness of interventions that affect fertility and childbearing:how health effects are measured matters. Med Decis Making. (2015) 35(7):818–46. 10.1177/0272989/15583845 25926281 PMC4418217

[64] Scotland G McLernon D Kurinczuk J McNamee P Harrild K Lyall H Minimising twins in in vitro fertilisation: a modelling study assessing the costs, consequences and cost–utility of elective single versus double embryo transfer over a 20-year time horizon. BJOG: Int J Obstet Gynaecol. (2011) 118(9):1073–83. 10.1111/j.1471-0528.2011.02966.x 21477172

[65] Busnelli A Papaleo E Del Prato D La Vecchia I Iachini E Paffoni A A retrospective evaluation of prognosis and cost-effectiveness of IVF in poor responders according to the Bologna criteria. Hum Reprod. (2014) 30(2):315–22. 10.1093/humrep/deu319 25432927

[66] Poulos TA. Modern art: determining a couple’s most cost-effective embryo transfer decision [honor’s thesis]. Durham: Duke University (2007).

[67] van Heesch MMJ van Asselt ADI Evers JLH van der Hoeven MAHBM Dumoulin JCM van Beijsterveldt CEM Cost-effectiveness of embryo transfer strategies: a decision analytic model using long-term costs and consequences of singletons and multiples born as a consequence of IVF. Hum Reprod. (2016) 31(11):2527–40. 10.1093/humrep/dew229 27907897

[68] Devlin N Parkin D. Funding fertility: issues in the allocation and distribution of resources to assisted reproduction technologies. Hum Fertil. (2003) 6:S2–6. 10.1080/1464770312331369153 12869768

[69] Botha W. Methodological challenges in economic evaluations of fertility treatments. Fertil Steril. (2018).

[70] ESHRE Capri Workshop Group, Baird DT Barri PN Bhattacharya S Devroey P Evers JLH Economic aspects of infertility care: a challenge for researchers and clinicians. Hum Reprod. (2015) 30(10):2243–8. 10.1093/humrep/dev163 26141712

[71] Torrance GW Feeny DH Furlong WJ Barr RD Zhang Y Wang Q. Multiattribute utility function for a comprehensive health Status classification system: health utilities Index mark 2. Med Care. (1996) 34(7):702–22. 10.1097/00005650-199607000-00004 8676608

[72] Luyten J Verbeke E Schokkaert E. To be or not to be: future lives in economic evaluation. Health Econ. (2022) 31(1):258–65. 10.1002/hec.4454 34743370

[73] Shih ST Farrar MA Wiley V Chambers G. Newborn screening for spinal muscular atrophy with disease-modifying therapies: a cost-effectiveness analysis. J Neurol Neurosurg Psychiatry Res. (2021) 92(12):1296–304. 10.1136/jnnp-2021-326344 34321343

[74] Shih STF Keller E Wiley V Farrar MA Wong M Chambers GM. Modelling the cost-effectiveness and budget impact of a newborn screening program for spinal muscular atrophy and severe combined immunodeficiency. Int J Neonatal Screen. (2022) 8(3):45. 10.3390/ijns8030045 35892475 PMC9326684

[75] Shih STF Keller E Wiley V Wong M Farrar MA Chambers GM. Economic evaluation of newborn screening for severe combined immunodeficiency. Int J Neonatal Screen. (2022) 8(3):44. 10.3390/ijns8030044 35892474 PMC9326549

[76] Shreffler KM Greil AL Tiemeyer SM McQuillan J. Is infertility resolution associated with a change in women’s well-being? Hum Reprod. (2020) 35(3):605–16. 10.1093/humrep/dez297 32112095 PMC7105324

[77] Feng Y-S Kohlmann T Janssen MF Buchholz I. Psychometric properties of the EQ-5D-5L: a systematic review of the literature. Qual Life Res. (2021) 30(3):647–73. 10.1007/s11136-020-02688-y 33284428 PMC7952346

[78] Rabin R de Charro F. EQ-SD: a measure of health status from the euroqol group. Ann Med. (2001) 33(5):337–43. 10.3109/07853890109002087 11491192

[79] Pettitt D Raza S Naughton B Roscoe A Ramakrishnan A Ali A The limitations of QALY: a literature review. J Stem Cell Res Ther. (2016) 6(4):1000334. 10.4172/2157-7633.1000334

[80] Norman R Olsen JA. Expanding the scope of value for economic evaluation: the EQ-HWB. Value Health. (2022) 25(4):480–1. 10.1016/j.jval.2022.02.001 35256244

[81] Basu A Sullivan SD. Toward a hedonic value framework in health care. Value Health. (2017) 20(2):261–5. 10.1016/j.jval.2016.11.022 28237206

[82] Neumann PJ Johannesson M. The willingness to pay for in vitro fertilization: a pilot study using contingent valuation. Med Care. (1994) 32(7):686–99. 10.1097/00005650-199407000-00003 8028404

[83] Keller E Newman JE Ortmann A Jorm LR Chambers GM. How much is a human life worth? A systematic review. Value Health. (2021) 24(10):1531–41. 10.1016/j.jval.2021.04.003 34593177

[84] Botha W Donnolley N Shanahan M Norman RJ Chambers GM. Societal preferences for fertility treatment in Australia: a stated preference discrete choice experiment. J Med Econ. (2019) 22(1):95–107. 10.1080/13696998.2018.1549055 30431385

[85] Cookson R Mirelman AJ. Equity in HTA: what doesn’t get measured, gets marginalised. Isr J Health Policy Res. (2017) 6(1):38. 10.1186/s13584-017-0162-3 28694961 PMC5502411

[86] National Institute for Health and Care Excellence (NICE). Chte methods review: health-related quality of life: task and finish group report (2020).

[87] National Institute for Health and Care Excellence (NICE). The guidelines manual: process and methods. London: NICE (2021).

[88] Drummond MF Neumann PJ Sullivan SD Fricke F-U Tunis S Dabbous O Analytic considerations in applying a general economic evaluation reference case to gene therapy. Value Health. (2019) 22(6):661–8. 10.1016/j.jval.2019.03.012 31198183

[89] Basu A Carlson J Veenstra D. Health years in total: a new health objective function for cost-effectiveness analysis. Value Health. (2020) 23(1):96–103. 10.1016/j.jval.2019.10.014 31952678

[90] Medical Services Advisory Committee. Technical guidelines for preparing assessment reports for the medical services advisory committee - service type: investigative (Version 3.0). Canberra: Health AGDo (2017).

[91] Lakdawalla DN Phelps CE. Health technology assessment with diminishing returns to health: the generalized risk-adjusted cost-effectiveness (grace) approach. Value Health. (2021) 24(2):244–9. 10.1016/j.jval.2020.10.003 33518031

[92] Turner HC Archer RA Downey LE Isaranuwatchai W Chalkidou K Jit M An Introduction to the main types of economic evaluations used for informing priority setting and resource allocation in healthcare: key features, uses, and limitations. Front Public Health. (2021) 9:1–17. 10.3389/fpubh.2021.722927 PMC8424074 34513790

[93] The Pharmaceutical Benefits Scheme (PBS). Pbs news: reference committee for the health technology assessment policy and methods review: commonwealth of Australia (2022). Available at: https://www.pbs.gov.au/info/news/2022/04/reference-committee-HTA-review (Accessed 10 April, 2022–22 August, 2022).

[94] Paulden M. Recent amendments to nice’s value-based assessment of health technologies: implicitly inequitable? Expert Rev Pharmacoecon Outcomes Res. (2017) 17(3):239–42. 10.1080/14737167.2017.1330152 28490259

[95] Boysen M. Health technology evaluation process. Methods and topic selection review: conclusions and final update. Centre for Health Technology Evaluation (2022).

[96] McIntosh E. Using discrete choice experiments within a cost-benefit analysis framework: some considerations. PharmacoEconomics. (2006) 24(9):855–68. 10.2165/00019053-200624090-00004 16942121

[97] Tinelli M Ryan M Bond C. What, who and when? Incorporating a discrete choice experiment into an economic evaluation. Health Econ Rev. (2016) 6(1):31. 10.1186/s13561-016-0108-4 27472943 PMC4967060

